# Loss of *Dip2b* leads to abnormal neural differentiation from mESCs

**DOI:** 10.1186/s13287-023-03482-6

**Published:** 2023-09-13

**Authors:** Mingze Yao, Yuanqing Pan, Tinglin Ren, Caiting Yang, Yu Lei, Xiaoyu Xing, Lei Zhang, Xiaogang Cui, Yaowu Zheng, Li Xing, Changxin Wu

**Affiliations:** Institutes of Biomedical Sciences, Shanxi Provincial Key Laboratory for Medical Molecular Cell Biology, Key Laboratory of Chemical Biology and Molecular Engineering of Ministry of Education, Shanxi University, Taiyuan, 030006 China

**Keywords:** *Dip2b*, Neural differentiation, Axon guidance, CRISPR/Cas9, Gene knockout, RNA-seq, mESCs

## Abstract

**Background:**

Disco-interacting protein 2 homolog B is a member of the Dip2 family encoded by the *Dip2b* gene. *Dip2b* is widely expressed in neuro-related tissues and is essential in axonal outgrowth during embryogenesis.

**Methods:**

*Dip2b* knockout mouse embryonic stem cell line was established by CRISPR/Cas9 gene-editing technology. The commercial kits were utilized to detect cell cycle and growth rate. Flow cytometry, qRT-PCR, immunofluorescence, and RNA-seq were employed for phenotype and molecular mechanism assessment.

**Results:**

Our results suggested that *Dip2b* is dispensable for the pluripotency maintenance of mESCs. *Dip2b* knockout could not alter the cell cycle and proliferation of mECSs, or the ability to differentiate into three germ layers in vitro. Furthermore, genes associated with axon guidance, channel activity, and synaptic membrane were significantly downregulated during neural differentiation upon *Dip2b* knockout.

**Conclusions:**

Our results suggest that *Dip2b* plays an important role in neural differentiation, which will provide a valuable model for studying the exact mechanisms of *Dip2b* during neural differentiation.

**Supplementary Information:**

The online version contains supplementary material available at 10.1186/s13287-023-03482-6.

## Introduction

*Dip2b* gene-encoded disconnected-interacting protein 2 homolog B (DIP2B), a member of the DIP2 protein family, is a highly conserved protein of about 1576 amino acids in length and contains a DMAP1-binding domain. DIP2B is involved in DNA methylation processes [1] and expressed in neuronal, myocardial, endothelial, and epithelial cells [2]. DIP2B plays critical roles in cell proliferation, migration, and apoptosis during embryogenesis [3], and lung maturation and survival [4], and also is linked to several neuro-related diseases including schizophrenia [5], bipolar disorder [6], and chorea [7].

Embryonic stem cells (ESCs) are pluripotent and can differentiate into neural precursor cells (NPCs) and subsequently into neurons under certain conditions [8]. The morphogenesis of a neuron is initiated when newborn neurons extend multiple neurites. Then, a single neurite elongates and develops into an axon, while the others extend slowly and differentiate into dendrites [9, 10]. During neuronal development, neurons project axons and dendrites to reach their cognate targets by modulating the cytoskeleton to establish neuronal networks [11–13]. DIP2B promotes axonal outgrowth by interacting with α-tubulin during early development of mice [14]. However, the function of DIP2B in neural differentiation of mESCs remains unknown.

To explore the role of DIP2B in neural induction in vitro, we used CRISPR/Cas9 gene-editing technology to establish *Dip2b* knockout mESCs cell line. The results demonstrated that the functional loss of *Dip2b* did not affect the pluripotency of mESCs or its ability to differentiate toward three germ layers in spontaneous embryoid body (EB) differentiation. *Dip2b* knockout did not affect cell proliferation and cell cycle, but led to dysregulation of axonogenesis, axon guidance, neuron projection guidance, focal adhesion, and ECM-receptor interaction pathways.

## Materials and methods

### Culture of mESCs

46C mESCs (*Sox1-gfp*) [15] were incubated in a humidified atmosphere containing 5% CO_2_ at 37 °C and cultured on gelatin-coated plates in Dulbecco’s modified Eagle High Glucose medium (DMEM, Hyclone) supplemented with 20% fetal bovine serum (FBS, SOFRA), 1 mM non-essential amino acids (Gibco), 1 × GlutaMAX (Gibco), 1 mM sodium pyruvate (Gibco), 50 units/mL penicillin and 50 μg/mL streptomycin (Hyclone), 0.1 mM β-mercaptoethanol, 1000 U/mL leukemia inhibitory factor (LIF, Sino Biological lnc), and 2i inhibitors (3 μM CHIR99021 and 1 μM PD0325901). For all experiments, the medium was refreshed every day. Once the cells were grown to 70% confluence, we passaged cells with trypsin containing 0.25% EDTA (Gibco) at a 1:5 split ratio.

### Generation of *Dip2b* knockout mESCs

Plasmids pMD-18T containing PGK-Puro-P2A-mCherry and pX330 containing the sgRNA sequence targeting the eighth exon of *Dip2b* were constructed. According to the manufacturer’s instructions (Ltd.P3 Primary Cell 4D-Nucleofector™ X Kit L (V4XP-3012), Lonza), two plasmids were mixed in proportion (3:1, 6 μg in total) and electroporated into 1 × 10^6^ 46C mESCs. The cells were subsequently screened in puromycin (1 μg/mL, Gibco)-containing medium. Fluorescence-activated cell sorting (FACS) (Beckman, MoFlo Astrios EQ) was used to isolate cells with significant fluorescence. After culturing through two passages, individual clones were selected when the cell density reached 60% and were plated on gelatin-coated 48-well plates (one clone per well). Then, we used Tiangen Genomic DNA Kit (TIANGEN) to extract genomic DNA and verified gene knockout by PCR (Genstar LongTaq PCR StarMix) on Veriti™ 96-Well Fast Thermal Cycler (Thermo Fisher) under the following conditions: initial denaturation at 95 °C for 3 min, 35 cycles of 95 °C for 15 s and 60 °C for 15 s and 72 °C for 30 s. Quantitative real-time PCR (qRT-PCR) and genomic PCR were performed to verify the depletion of *Dip2b* mRNA and the genomic sequence of exon 8. The primers used in the PCR assays are listed in Additional file 1: Table S5.

### RNA isolation and qRT-PCR

Total RNA was extracted using the EZ-press RNA Purification Kit (EZBioscience), then HiScript® III RT SuperMix for qPCR (Vazyme) was used to produce cDNA. qRT-PCR in triplicate using 2 × RealStar Green Power Mixture (GenStar) on CFX Connect (Bio-Rad) was performed under the following conditions: initial denaturation at 95 °C for 10 min, 40 cycles of 95 °C for 10 s and 60 °C for 10 s and 72 °C for 20 s. Primers used for the qRT-PCR are listed in Additional file 1: Table S6.

### Immunofluorescence staining

Cells were washed twice with PBS and fixed with 4% paraformaldehyde for 20 min at room temperature. After being washed twice again, cells were permeabilized with 0.3% TritonX-100 for 10 min, blocked with 5% bovine serum albumin (BSA) at 37 °C for 1 h, and then incubated with primary antibodies overnight at 4 °C. After washing three times for 5 min each wash, cells were stained with secondary antibodies for 2 h in dark at room temperature. Subsequently, cells were washed three times, and 4′,6-diamidino-2-phenylindole (DAPI) (1 μg/mL for 10 min) was added to stain cell nuclei. The cells were washed three times and captured with a Confocal laser scanning microscope (Zeiss LSM 710). The antibodies used in immunofluorescence staining include: anti-OCT4 (1:200, Santa Cruz biotechnology, USA, Cat#sc-5279), anti-NANOG (1:500, Novus Biologicals, USA, Cat#NB100-58842), anti-PAX6 (1:250, Proteintech, China, Cat#12323-1-AP), anti-GFP (1:100, Proteintech, China, Cat#50430-2-AP), and anti-*β-III Tubulin* (1:200, ABclonal, China, Cat#A18132).

### Embryoid body (EB) formation

Hanging drops (1000/2000 cells per drop) were used to culture cells to form EBs in a differentiation medium. The medium was prepared without LIF and 2i inhibitors. After 4 days, the EBs were transferred into 100 mm Petri dishes in a differentiation medium for 3 days. Total RNA was then extracted, and the expression of three germ marker genes were subsequently detected by qRT-PCR. All experiments were repeated for at least three times.

### Mycoplasma detection

To confirm if the cells are mycoplasma positive or negative, this assay was performed using MycoAlert™ PLUS Mycoplasma Detection Kit (Lonza). One milliliter of cell supernatant was collected and centrifuged at 250 g for 5 min. Then, a 100-μL sample was resuspended in the same volume of MycoAlert PLUS Reagent in a luminometer plate, and A value was noted after 5 min of incubation. Similarly, for the B value, a 100-μL of MycoAlert™ PLUS Substrate was added to the sample and measured after 10 min of incubation. Finally, we calculated the ratio of the two values (B/A < 1), indicating no mycoplasma in the sample.

### Karyotype analysis

The 46C-*Dip2b* KO cell line (KO), after 12 passages, was analyzed for karyotyping by Guangzhou Chunshui Bioscience & Technology Co. A total of 20 metaphase spreads were randomly selected and analyzed.

### STR analysis

STR analysis was performed on the 46C-*Dip2b* KO cell line and wild-type 46C (WT) with the detection of nine loci (4–2, 5–5, 6–4, 6–7, 9–2, 12–1, 15–3, 18–3, X-1) (Sangon Biotech Co., Ltd., Shanghai, China).

### Monolayer neural differentiation

Monolayer neural differentiation was performed according to a previous report [16]. WT and KO mESCs were plated in 12-well plates with 40,000 cells in N2B27 neural differentiated medium containing 50% DMEM/F12 (Sevenbio, Beijing, China), 50% Neurobasal (Gibco), 0.5% N2 (Gibco), 1% B27 (Gibco), 1 × GlutaMAX (Gibco), 1 mM NEAA (Gibco), and 0.1 mM β-mercaptoethanol. The medium was changed every day. The cells were collected for qRT-PCR assay at days 0, 4, and 8.

### Telencephalic differentiation

Telencephalic differentiation was performed as reported previously [17]. WT and KO mESCs were plated in 6 cm-diameter plates with 250,000 cells in KSR differentiated medium (GMEM (Gibco) containing 7% knockout serum replacement (Gibco), 1 × GlutaMAX, 1 mM NEAA, 1 mM Sodium pyruvate, and 0.1 mM β-mercaptoethanol). The medium was changed every 2 days. The cells were collected for qRT-PCR assay on day 0, day 4, and day 9.

### CCK8 proliferation assay

Cell proliferation was assessed by performing CCK8 experiment with a Cell Counting Kit-8 (Beyotime, China, C0037) according to the instruction of manufactory. 2000 WT and *Dip2b* KO cells were cultured on a 96-well culture plate. At days 1, 2, 3, 4, and 5, 10 μL CCK8 was added into 100 μL culture medium at 37 °C for 2 h. The absorbance was measured at 450 nm using a multifunctional microporous detector.

### Cell cycle analysis

60,000 WT and KO mESCs were plated in 6-well plates for 3.5 days. Cells were collected and washed twice with DPBS, and about 10,000 cells were fixed in 70% ethanol at 4 °C overnight. Cells were then centrifuged and washed with DPBS, suspended in 0.5 mL propidium iodide (PI) staining solution, and incubated at 37 °C for 30 min in dark. A BD flow cytometer (BD Biosciences) was used to measure cell cycle progression.

### Flow cytometry

*Sox1*-GFP was used to evaluate the efficiency of neural stem cell generation [15]. The cells were harvested by Trypsin–EDTA and Accutase, and then washed with 500 μL DPBS. Flow cytometry was performed using a BD flow cytometer (BD Biosciences).

### Bulk RNA-seq data analysis

Raw reads were quality-checked with fastp v 0.20.1 [18], an ultra-fast FASTQ preprocessor. Kallisto index was created based on the GRCm38.p6 and RefSeq transcriptome. Next, Kallisto v 0.44.0 was run to align paired-end reads directly to this index and estimate expression levels in transcripts per million (TPM) based on the alignments [19]. To determine differentially expressed genes (DEGs), the low abundance genes with low counts were first discarded, and then the remaining genes were conducted with edgeR (biological coefficient of variation set at 0.1) to obtain significant DEGs by setting padj < 0.05 and log2(fold change) ≥ 1. After that, the functional enrichment analysis of GO, KEGG and Gene Set Enrichment Analysis (GSEA) were performed by R package clusterProfiler.

## Results

### Establishing mESCs with functional loss of *Dip2b*

**Fig. 1:**
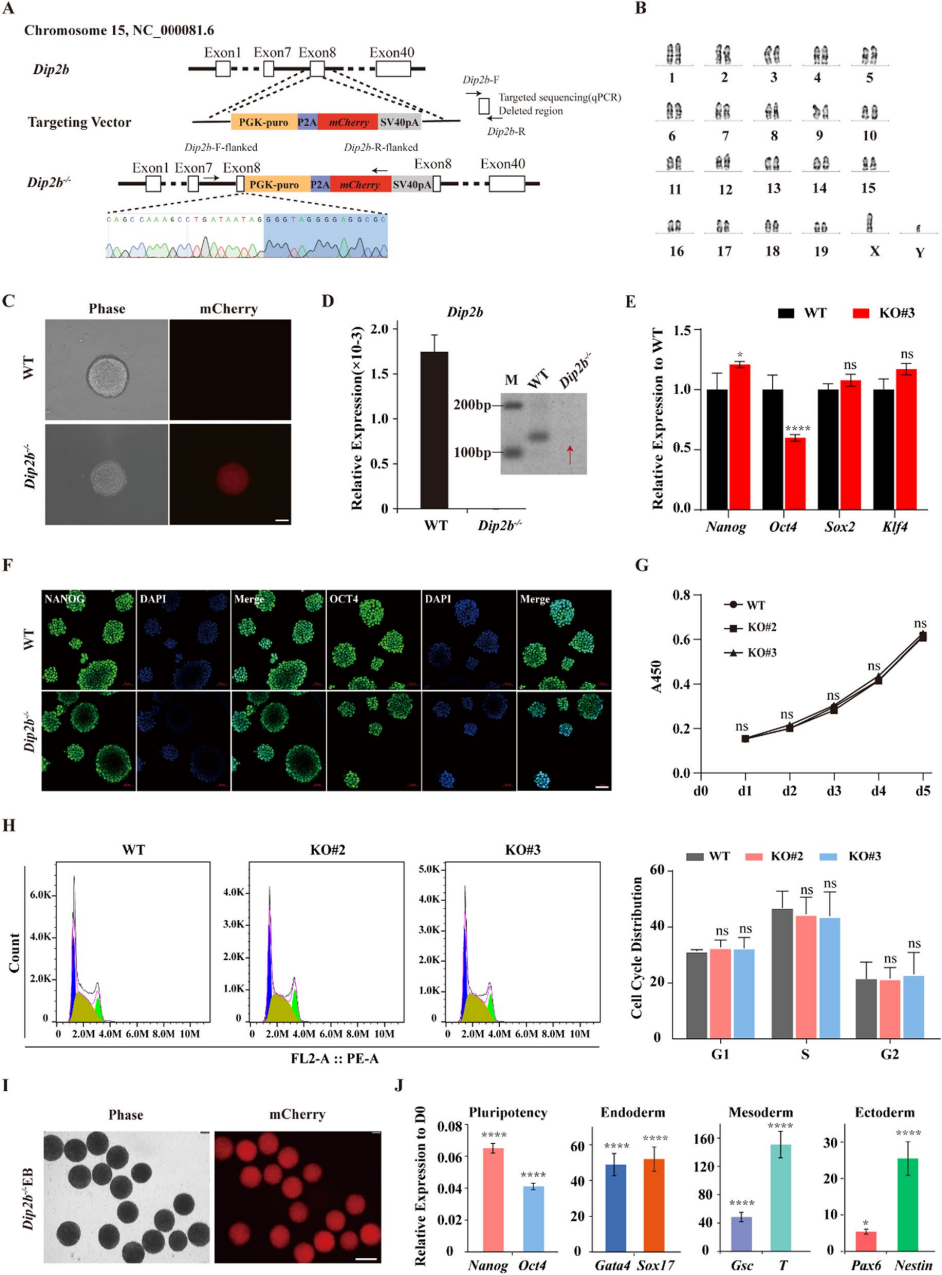
*Dip2b* is dispensable for pluripotency maintenance of mouse ESCs. **A** Schematic of *Dip2b* knockout. **B** Karyotype analysis of KO ESCs after 12 passages. **C** Representative morphological images of WT and KO clones (*n* = 3). Scale bar, 25 μm. **D** qRT-PCR (left) and PCR (right, full-length gels are presented in Additional file 2) of *Dip2b* expression in WT and *Dip2b* knockout (*n* = 3). **E** qRT-PCR of representative pluripotent genes expression in WT and KO ESCs (*n* = 3). **F** Representative immunofluorescence staining of pluripotent marker NANOG and OCT4 for WT and KO ESCs (*n* = 3). Scale bar, 50 μm. **G** A450 for CCK8 assay in three time points during culture (*n* = 3). **H** Cell cycle analysis using PI staining in WT and KO ESCs. The populations of different phases were calculated in FlowJo (v10.8.1) (*n* = 3). **I** Representative morphology of EB formation derived from KO ESC expressing mCherry (*n* = 3). Scale bar, 25 μm. **J** qRT-PCR of representative pluripotent and lineage genes expression at day 6 post differentiation in KO ESCs (*n* = 3). The error bars represented the mean ± SD, and the significance level was calculated by Student’s *t* test (two-tailed, equal variance) (ns, not statistically significant; *P < 0.05, **P < 0.01, ***P < 0.001, ****P < 0.0001)

To investigate the biological function of *Dip2b* during the neural differentiation process of mESCs, we established a *Dip2b* homozygous knockout 46C ES cell line using the CRISPR/Cas9 genome editing technology, which expresses GFP under control of the Sox1 promoter (*Sox1-gfp* mESCs) [15]. A targeting vector consisting of a 1206-bp left arm, PGK-Puro, P2A-mCherry and a 1201-bp right arm and a sgRNA-PX330 plasmid were co-electroporated into 46C mESCs. The Cas9 enzyme, specifically guided by the sgRNA, can cleave the eighth exon of *Dip2b* to produce a double-strand break. The eighth exon of *Dip2b* was then replaced with PGK-Puro-P2A-mCherry via homologous recombination (Fig. 1A). As shown in Fig. 1B, the 46C-*Dip2b* knockout cell line showed a normal karyotype, a typical stem cell morphology, and expressed mCherry (Fig. 1C). To confirm that the *Dip2b* gene knockout cell line is generated successfully, the deletion region in both DNA and RNA was examined. Deletion region was not detectable at DNA level by PCR (Fig. 1A, D, right), or at RNA level by qRT-PCR (Fig. 1A, D, left), confirming the successful deletion in the expected region of *Dip2b* via CRISPR/Cas9-mediated donor insertion (Fig. 1A and Additional file 1: Fig. S1).

We then assessed whether *Dip2b* knockout affects the expression of pluripotent marker proteins. The qRT-PCR analysis of pluripotent genes revealed that mRNAs of *Nanog*, *Oct4*, *Sox2*, and *Klf4* in the KO cell line were at the similar level to that of WT (Fig. 1E), indicating that *Dip2b* is not required for the maintenance of mESC pluripotency. Immunofluorescence staining results further confirmed that OCT4 and NANOG in the WT and KO groups were comparable (Fig. 1F), indicating that *Dip2b* is dispensable for the pluripotency maintenance of mESCs.

Since *Dip2b* can reduce cell proliferation and increase apoptosis in *Dip2b*-deficient MELFs [3], we examined the cell cycle and growth rate of the KO ESCs by performing CCK8 assay and found that the proliferation rate of KO ESCs was similar to that of WT (Fig. 1G). The PI staining assay further confirmed that the cell cycle progression was not altered by *Dip2b* knockout (Fig. 1H). Since the ability to form EBs in vitro is inherent to all pluripotent stem cells [20], which mimic early embryogenesis, we measured the three-layer markers to examine the spontaneous differentiation ability toward three layers only in KO mESCs (Fig. 1I). Intriguingly, the expression of all the three germ layer genes (*Pax6* and *Nestin* for ectoderm; *Gata4* and *Sox17* for endoderm, *Gsc* and *T* for mesoderm) was significantly upregulated (Fig. 1J). These results demonstrate that *Dip2b* knockout can not alter the cell cycle and proliferation of mECSs or the ability to differentiate into three germ layers in vitro.

Next, we verified four off-target sites based on the Zhang laboratory website (https://zlab.bio/guide-design-resources), and no off-target event was detected from our designed sgRNA. No mycoplasma was detected in the cell line. STR analysis proved that the KO cell line and WT were identical to the parent cell line.

### *Dip2b* is involved in the rosette organization of the neural precursor clusters

**Fig. 2:**
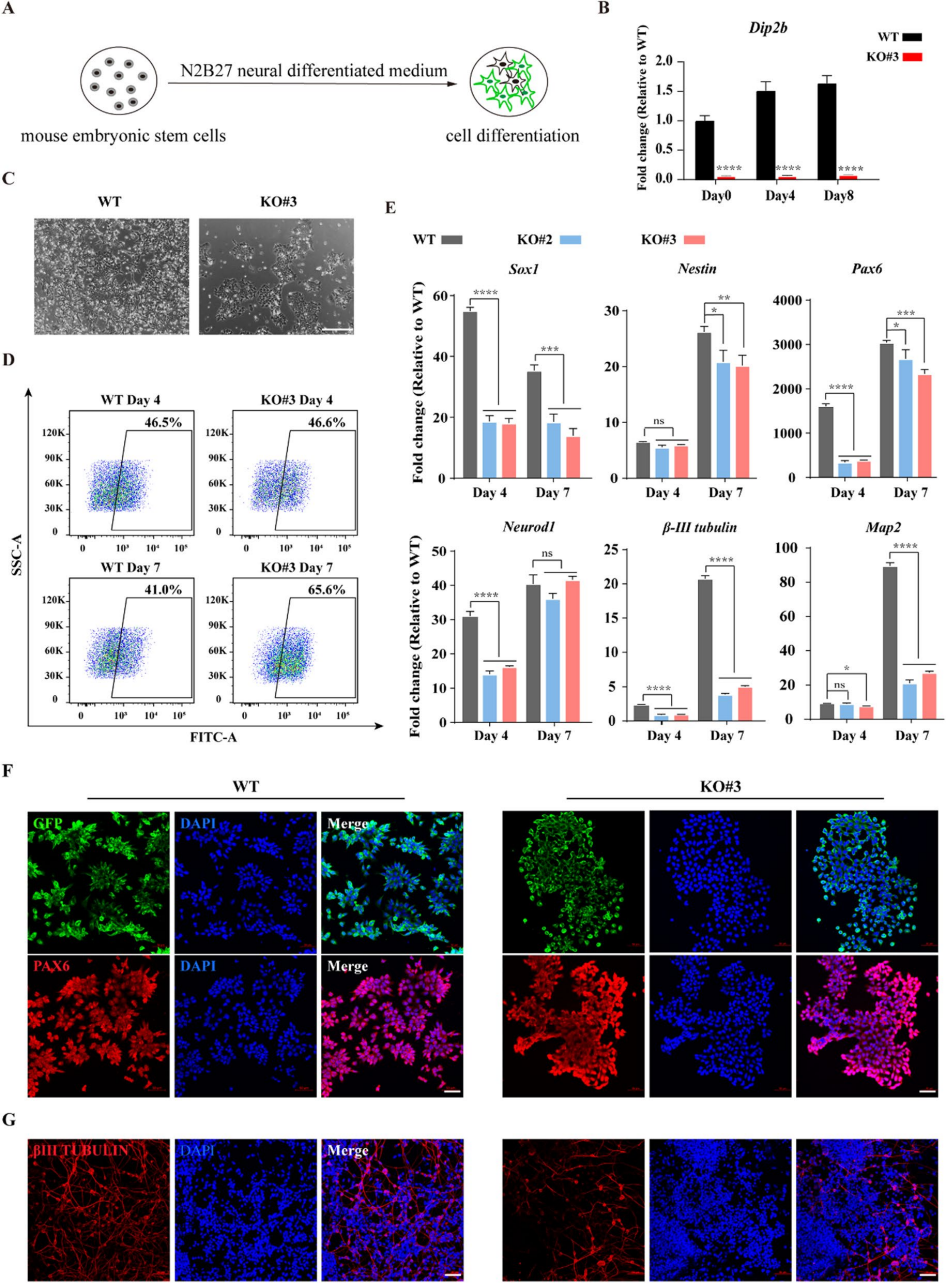
*Dip2b* loss-of-function disrupts neural differentiation in the N2B27 medium. **A** Schematic diagram showing differentiation of mESCs in N2B27 medium. **B** qRT-PCR of *Dip2b* expression at days 0, 4, 8 post differentiation (*n* = 3). **C** Representative morphological images of WT and KO cells at day 3 post differentiation (*n* = 3). Scale bar, 250 μm. **D** Flow cytometric analysis showing the percentage of Sox1-GFP positive cells in differentiated WT and KO cells (*n* = 3). **E** Expression of marker genes by qRT-PCR in WT and KO cells at days 4 and 7 post differentiation (*n* = 3). **F** and **G** Immunofluorescence staining of WT and KO cells at days 4 (**F**) and 10 (**G**) post differentiation (adherent culture on dishes coated with poly-D-lysine and laminin, *n* = 3). Scale bars, 50 μm. The error bars represented the mean ± SD, and the significance level was calculated by Student’s *t* test (two-tailed, equal variance) (ns, not statistically significant; *P < 0.05, **P < 0.01, ***P < 0.001, ****P < 0.0001)

To investigate whether *Dip2b* is involved in neural differentiation, mESCs were subjected to monolayer differentiation toward neural lineage in N2B27 medium (Fig. 2A). The expression of *Dip2b* was gradually increased in WT cells compared to KO cells during the neural differentiation (Fig. 2B), indicating *Dip2b* might have functions on this process. Because N2B27 medium could support the long-term maintenance and maturation of neuronal cells [8], we observed a similar rosette organization of the neural precursor clusters as known previous research [16, 21] at day 3 post differentiation in WT mESCs (Fig. 2C). FACS analysis showed a significantly higher percentage of Sox1-GFP positive cells in differentiated KO cells at later neuronal differentiation stage (Fig. 2D), which was inconsistent with the results of qRT-PCR, possibly due to abnormal protein level consumption after *Dip2b* knockout. This may lead to repression of neural precursor to differentiate toward neurons. The qRT-PCR results showed that the mRNAs level of neural stem cell markers (*Sox1*, *Pax6* and *Nestin*), early neuronal markers (*β-III tubulin* and *Neurod1*), and mature neuronal marker (*Map2*) were all significantly downregulated in KO mESCs (Fig. 2E). Furthermore, immunofluorescence results showed that WT cells had more typical rosette organizations in the early stages of differentiation compared to KO differentiated cells (Fig. 2F), although the fluorescence intensity of the neural stem cell markers (GFP-SOX1, PAX6) was at the comparable level. Notably, WT cells showed stronger expression of neuronal marker β-III TUBULIN at later differentiation stage compared to KO cells (Fig. 2G). These results suggest that *Dip2b* loss-of-function leads to abnormal differentiation of neural precursors cells, thus impeding the neuronal differentiation.

### *Dip2b* affects the conversion of mESCs into neural fates in suspension culture

**Fig. 3:**
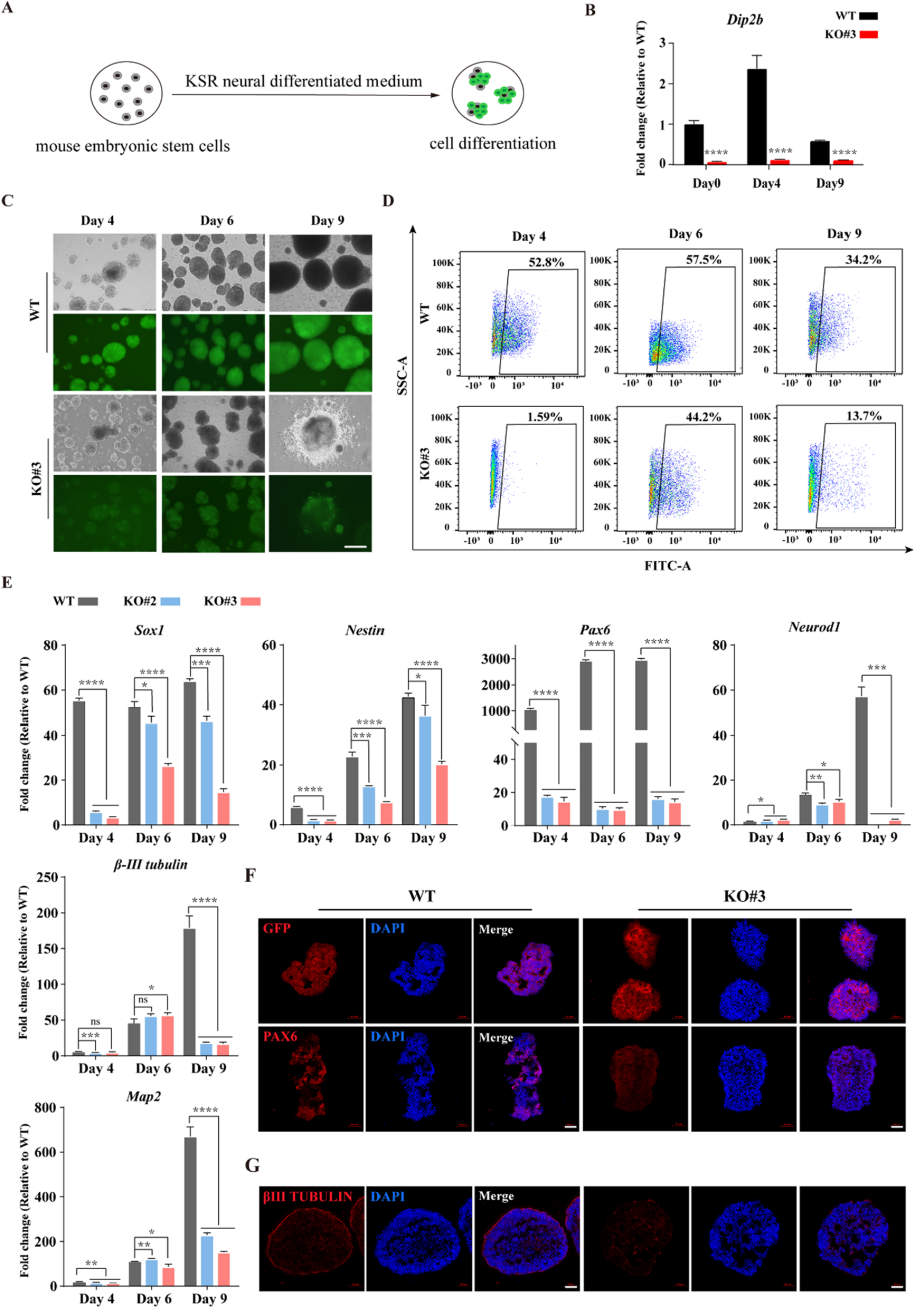
*Dip2b* loss-of-function disrupts neural differentiation in the KSR medium. **A** Schematic diagram showing differentiation of mESCs in KSR medium. **B** qRT-PCR of *Dip2b* expression at days 0, 4, and 9 post differentiation (*n* = 3). **C** Representative morphological images of WT and KO cells at days 4, 6, and 9 post differentiation (*n* = 3). Scale bar, 200 μm. **D** Flow cytometric analysis showing the percentage of Sox1-GFP positive cells in differentiated WT and KO cells (*n* = 3). **E** Expression of marker genes by qRT-PCR in WT and KO cells at days 4, 6, and 9 post differentiation (*n* = 3). **F** and **G** Immunofluorescence staining of WT and KO cells at days 6 (**F**) and 10 (**G**) post differentiation (*n* = 3). Scale bars, 50 μm. The error bars represented the mean ± SD, and the significance level was calculated by Student’s *t* test (two-tailed, equal variance) (ns, not statistically significant, *P < 0.05, **P < 0.01, ***P < 0.001, ****P < 0.0001)

To further determine whether *Dip2b* is required for neural differentiation, we used a KSR medium to induce KO and WT cells toward telencephalic precursors (Fig. 3A). We then collected differentiated cells on day 4 and day 9 to test the expression levels of *Dip2b* and found that the expression of *Dip2b* gene was initially increased and then decreased in WT cells compared to KO cells during the neural differentiation, suggesting the importance of *Dip2b* during early neural differentiation (Fig. 3B). We also observed that KO cells had weaker fluorescence intensity and non-smooth morphology than WT in time course differentiation experiments (Fig. 3C). Consistent with these results, flow cytometric analysis of Sox1-GFP further confirmed that *Dip2b* was required for neural differentiation (Fig. 3D). Moreover, qRT-PCR results showed that the expression of neural progenitor markers, early neuronal markers, and a mature neuronal marker were dramatically reduced at the mRNA level in KO cells compared to WT group (Fig. 3E). In addition, immunofluorescence results showed that WT cells showed stronger expression of PAX6 in the early stage of differentiation (Fig. 3F), which was consistent with the results of qRT-PCR. Similar to N2B27 differentiation, WT cells showed stronger expression of a neuron marker β-III TUBULIN during late differentiation (Fig. 3G). These results together indicate that *Dip2b* is vital for converting mESCs to neural fates in suspension culture.

### *Dip2b* knockout depresses axonogenesis and axon guidance

**Fig. 4:**
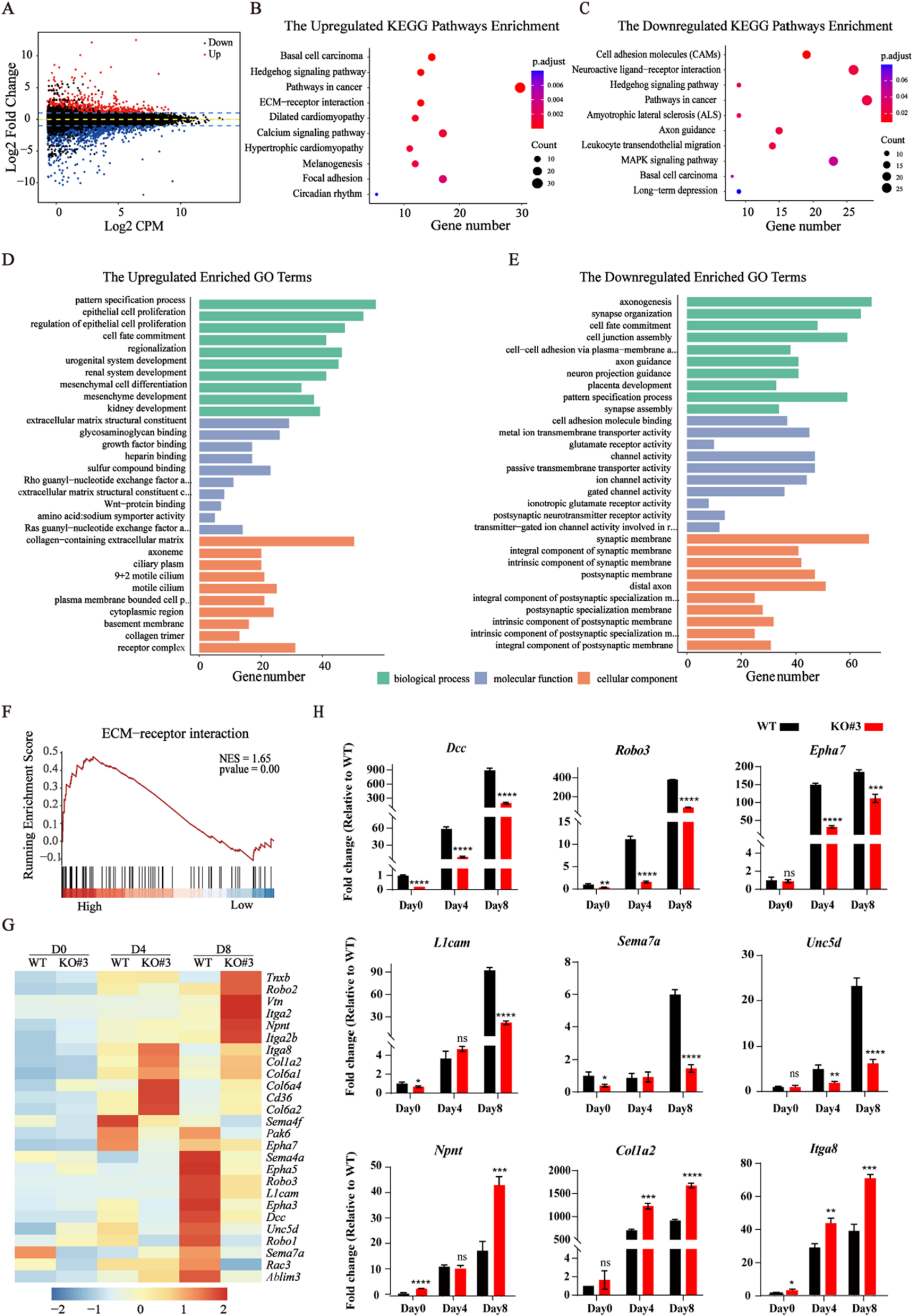
*Dip2b* knockout depresses axon guidance in N2B27 medium. **A** MA plot showing the expression of DEGs from KO versus WT mESCs at day 8 post differentiation. Upregulated and downregulated genes are plotted in red and blue, respectively. **B** and **C** KEGG pathway analysis of DEGs from KO versus WT at day 8 post differentiation. **D** and **E** GO analysis. Top ten GO terms of upregulated and downregulated DEGs from KO versus WT at day 8 post differentiation. **F** The GSEA showing the expression pattern of ECM-receptor interaction-related genes in KO and WT. **G** Heatmap showing the expression of DEGs in KO and WT. **H** Expression of upregulated and downregulated genes by qRT-PCR in WT and KO cells (*n* = 3). Values represent mean ± SEM. (ns, not statistically significant, *P < 0.05, **P < 0.01, ***P < 0.001, ****P < 0.0001)

To characterize the transcriptomic aberrations during neural differentiation of KO cells compared with WT cells, we performed RNA-seq at days 4 and 8 post differentiation under N2B27 differentiation condition, respectively. The RNA-seq results showed that 2175 genes were differentially expressed at day 8 post differentiation between KO versus WT, including 936 upregulated genes and 1239 downregulated genes (Fig. 4A). 704 upregulated genes and 499 downregulated genes were differentially expressed at day 4 post differentiation (Additional file 1: Fig. S2A). Twenty of most DEGs were listed in Additional file 1: Tables S1 and S2.

To elucidate the potential biological pathways under *Dip2b* regulation, we performed the KEGG pathway analysis. KEGG classification revealed that ‘Basal cell carcinoma’ and ‘Hedgehog signaling pathway’ were enriched in upregulated pathways (Fig. 4B and Additional file 1: Fig. S2B), whereas neuron- and synapse-related pathways including ‘Cell adhesion molecules (CAMs)’, ‘neuroactive ligand-receptor interaction’, and ‘Axon guidance’ were enriched in downregulated genes (Fig. 4C and Additional file 1: Fig. S2C).

To identify the potential biological roles of *Dip2b* in the neural differentiation process, DEGs identified from the comparison of KO and WT cells were used for gene ontology (GO) analysis. For the upregulated genes, top 10 most enriched terms were highly enriched as shown in Fig. 4D and Additional file 1: Fig. S2D.

For the downregulated genes, GO analysis revealed that annotated BP categories included ‘axonogenesis’, ‘synapse organization’, ‘axon guidance’, and ‘cell fate commitment’. MF categories included ‘cell adhesion molecular binding’, ‘metal ion transmembrane transporter activity’, and ‘channel activity’. CC categories included ‘synaptic membrane’, ‘postsynaptic membrane’, and ‘distal axon’ (Fig. 4E and Additional file 1: Fig. S2E). These results revealed that cluster terms from BP, MF, and CC categories were mainly enriched with DEG related to the axon, membrane structure and membrane activities.

To understand how *Dip2b* knockout represses neural differentiation, we performed a comprehensive transcriptome analysis. The GSEA revealed that most downregulated genes were highly enrichment in ECM-receptor interaction in KO cells (Fig. 4F and Additional file 1: Fig. S2F). The ECM in the CNS is known to be associated with the regulation of synaptic plasticity and various pathophysiological processes [22]. Many genes were activated during neural differentiation, while most of these genes failed to be activated or its expression was significantly reduced upon *Dip2b* knockout (Fig. 4G). We also validated these genes using qRT-PCR. As expected, qRT-PCR results were highly consistent with the RNA-seq data (Fig. 4H). Altogether, these data demonstrate that loss of *Dip2b* leads to the inhibition of axonogenesis-related genes during neural differentiation.

### *Dip2b* knockout disrupts ECM-receptor interaction and axon guidance

**Fig. 5:**
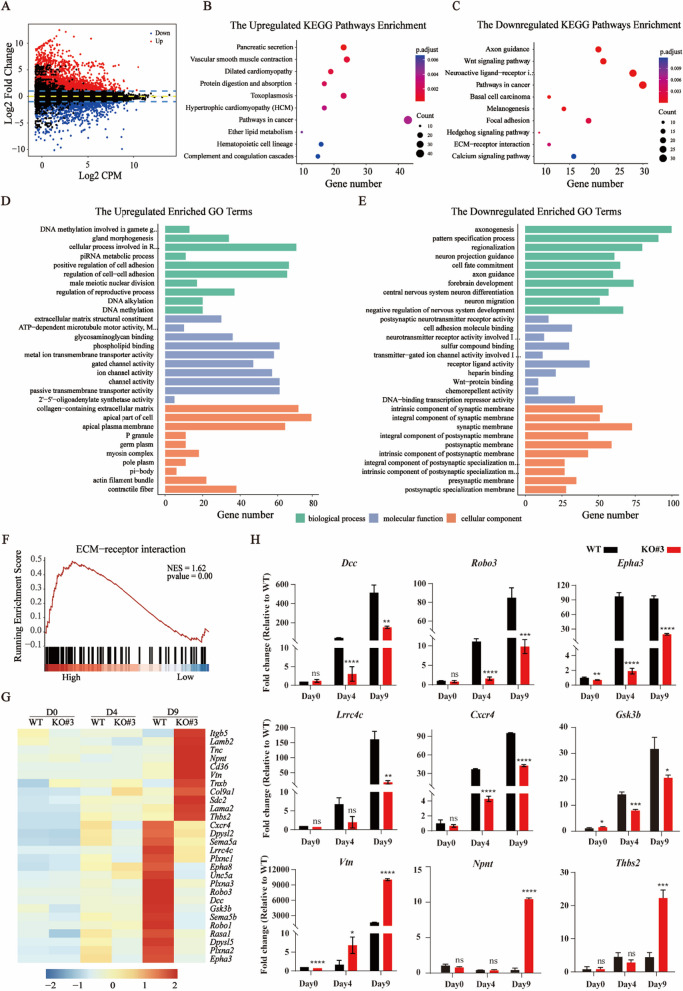
*Dip2b* knockout affects ECM-receptor interaction and axon guidance in the KSR medium. **A** MA plot showing the expression of DEGs for KO versus WT at day 9 post differentiation. Upregulated and downregulated genes are plotted in red and blue, respectively. **B** and **C** KEGG pathway analysis of DEGs from KO versus WT at day 9 post differentiation. **D** and **E** GO analysis. Top ten GO terms of upregulated and downregulated DEGs from KO versus WT at day 9 post differentiation. **F** The GSEA showing the expression pattern of ECM-receptor interaction-related genes in KO and WT. **G** Heatmap showing the expression of DEGs in KO and WT. **H** Expression of upregulated and downregulated genes by qRT-PCR in WT and KO ESCs (*n* = 3). Values represent mean ± SEM (ns means not statistically significant, *P < 0.05, **P < 0.01, ***P < 0.001, ****P < 0.0001)

We also analyzed transcriptomes of KO cells and WT cells at days 4 and 9 post differentiation under the KSR differentiation condition, respectively. The RNA-seq analysis showed that 6249 genes were differentially expressed at day 9 post differentiation between KO versus WT, including 3476 upregulated genes and 2773 downregulated genes (Fig. 5A). 2349 upregulated genes and 1074 downregulated genes were differentially expressed at day 4 post differentiation (Additional file 1: Fig. S3A). Twenty of most upregulated and downregulated DEGs were listed in Additional file 1: Tables S3 and S4.

Next, we performed the KEGG pathway analysis, which revealed that ‘ECM-receptor interaction’ (Additional file 1: Fig. S3B) and ‘Pancreatic secretion’ (Fig. 5B) were enriched in upregulated pathways at days 4 and 9 post differentiation, respectively. However, ‘neuroactive ligand-receptor interaction’ and ‘Axon guidance’ were enriched in downregulated genes (Fig. 5C and Additional file 1: Fig. S3C).

For the upregulated genes, the top ten most enriched terms were shown in Fig. 5D and Additional file 1: Fig. S3D. For the downregulated genes, GO analysis showed that annotated BP, CC and MF categories are similar to N2B27 differentiation (Fig. 5E and Additional file 1: Fig. S3E). These results further revealed that *Dip2b* knockout led to dysregulation of axonogenesis, axon guidance, neuron projection guidance, focal adhesion, and ECM-receptor interaction pathways. The GSEA revealed that genes of KO cells were highly enrichment in ECM-receptor interaction (Fig. 5F) while genes of WT cells were highly enriched in axon guidance (Additional file 1: Fig. S3F). Similarly, at day 9 post differentiation between KO versus WT, among the upregulated genes, associated with ECM-receptor interaction was enriched, and among the downregulated genes, associated with axon guidance was enriched (Fig. 5G). Consistently, qRT-PCR results further confirmed the observed phenotype (Fig. 5H). Altogether, these results suggest that loss of *Dip2b* affects ECM-receptor interaction and axon guidance.

## Discussion

**Fig. 6:**
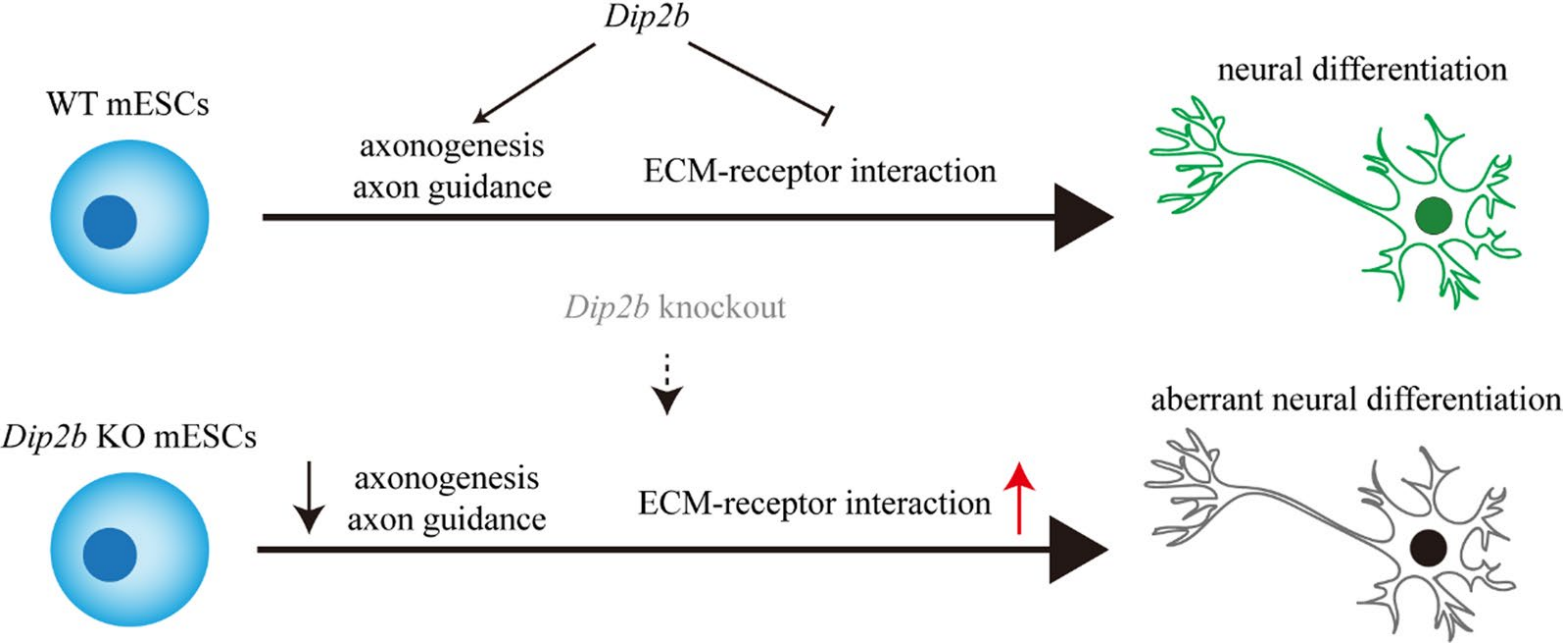
The roles of *Dip2b* during neural differentiation

In this study, by using a *Dip2b* knockout model, we reveal that *Dip2b* knockout affects neural differentiation of mESCs by depressing axonogenesis and axon guidance and facilitating ECM-receptor interaction (Fig. 6). *Dip2b* knockout cells retain the capacity to maintain self-renewal and differentiate into three germ layers. In contrast to the previous work demonstrating that *Dip2b* might regulate cell proliferation and differentiation [1, 3], our research shows that *Dip2b* knockout does not alter cell cycle and proliferation in mESCs.

In the mouse model, *Dip2b* expression is specific to the developing nervous system during the postgastrulation stages of mouse embryonic development [23]. In the neural differentiation assay, our results of monolayer differentiation show that *Dip2b* knockout cells failed to generate neural rosette organization, indicating that *Dip2b* inhibits the formation of NPCs. In suspension culture, we observed that *Dip2b* knockout had weaker fluorescence intensity and non-smooth morphology than WT in time course differentiation experiments. Both methods confirmed that *Dip2b* affects mESCs differentiation to neural fates. DIP2B knockout mice have abundant axonal development and synaptic transmission defects [14]. ECM is an important component in the microenvironment, which plays an important role in neuron maturation, communication, and aging [24]. Our RNA-seq results indicate that *Dip2b* knockout leads to dysregulation of axonogenesis, axon guidance, neuroactive ligand-receptor interaction, focal adhesion, and ECM-receptor interaction pathways.

## Conclusions

In summary, our study showed that *Dip2b* is critical for axonal development during the conversion of mESCs to neural fates. Based on our data, we speculate that during early developmental stages, the loss of *Dip2b* function inhibits axonogenesis, axon guidance, and neuron projection guidance.

### Supplementary Information

**Additional file 1.** **Fig. S1.** Verification of site-specific recombination by sequencing. A targeting vector consisting of a 1206-bp left arm, PGK-Puro, P2A-mCherry and a 1201-bp right arm and a sgRNA-PX330 plasmid were co-electroporated into 46C mESCs. The Cas9 enzyme, specifically guided by the sgRNA, can cleave the eighth exon of Dip2b to produce a double-strand break. The eighth exon of Dip2b was replaced with PGK-Puro-P2A-mCherry via homologous recombination. **Fig. S2.** *Dip2b* knockout depresses axon guidance in N2B27 medium. A MA plot showing the expression of DEGs from KO vs. WT at day 4 post differentiation. Upregulated and downregulated genes are plotted in red and blue, respectively. B and C KEGG pathway analysis of DEGs from KO vs. WT at day 4 post differentiation. D and E GO analysis. Top ten GO terms of upregulated and downregulated DEGs from KO vs. WT at day 4 post differentiation. F The GSEA showing the expression pattern of ECM-receptor interaction-related genes in KO and WT. **Fig. S3.** *Dip2b* knockout depresses axon guidance in KSR medium. A MA plot showing the expression of DEGs from KO vs. WT at day 4 post differentiation. Upregulated and downregulated genes are plotted in red and blue, respectively. B and C KEGG pathway analysis of DEGs from KO vs. WT at day 4 post differentiation. D and E GO analysis. Top ten GO terms of upregulated and downregulated DEGs from KO vs. WT at day 4 post differentiation. F The GSEA showing the expression pattern of axon guidance-related genes in KO and WT. **Table S1.** The 20 most differentially expressed genes between KO vs. WT at day 8 post differentiation under N2B27 differentiation condition (Fold change ≥ 2, padj < 0.05). **Table S2.** The 20 most differentially expressed genes between KO vs. WT at day 4 post differentiation under N2B27 differentiation condition (Fold change ≥ 2, padj < 0.05). **Table S3**. The 20 most differentially expressed genes between KO vs. WT at day 9 post differentiation under KSR differentiation condition (Fold change ≥ 2, padj < 0.05). **Table S4.** The 20 most differentially expressed genes between KO vs. WT at day 4 post differentiation under KSR differentiation condition (Fold change ≥ 2, padj < 0.05). **Table S5.** PCR primer sequences selected for validation. **Tables S6.** qRT-PCR primer sequences selected for validation.

**Additional file 2.** Uncropped gel images. Uncropped gel images are attached. Images used in the main figure are marked in red squares. Deletion region was not detectable at DNA level by PCR in the *Dip2b* gene knockout cell line.

## Data Availability

All data generated or analyzed during this study are included in this published article. The data of RNA-seq had been submitted to the NCBI Gene Expression Omnibus (GEO; https://www.ncbi.nlm.nih.gov/geo/) under accession number GSE232532.

## Abbreviations

DIP2B: Dip2p gene-encoded disconnected-interacting protein 2 homolog B
ESCs: Embryonic stem cells
NPCs: Neural precursor cells
EB: Embryoid body
BSA: Bovine serum albumin
DAPI: 4′,6-Diamidino-2-phenylindole
KO: 46C-*Dip2b* KO cell line
WT: Wild-type 46C
TPM: Transcripts per million
DEGs: Differentially expressed genes
GSEA: Gene set enrichment analysis
qRT-PCR: Quantitative real-time PCR
CAMs: Cell adhesion molecules
GO: Gene ontology
