# Supplementary material for: Loss of Dip2b leads to abnormal neural differentiation from mESCs
Source: Stem Cell Res Ther. 2023 Sep 13;14:248. doi: 10.1186/s13287-023-03482-6 (PMC10500737; doi:10.1186/s13287-023-03482-6)
Supplement: Supplementary file 1 — Additional file 1. Fig. S1. Verification of site-specific recombination by sequencing. A targeting vector consisting of a 1206-bp left arm, PGK-Puro, P2A-mCherry and a 1201-bp right arm and a sgRNA-PX330 plasmid were co-electroporated into 46C mESCs. The Cas9 enzyme, specifically guided by the sgRNA, can cleave the eighth exon of Dip2b to produce a double-strand break. The eighth exon of Dip2b was replaced with PGK-Puro-P2A-mCherry via homologous recombination. Fig. S2. Dip2b knockout depresses axon guidance in N2B27 medium. A MA plot showing the expression of DEGs from KO vs. WT at day 4 post differentiation. Upregulated and downregulated genes are plotted in red and blue, respectively. B and C KEGG pathway analysis of DEGs from KO vs. WT at day 4 post differentiation. D and E GO analysis. Top ten GO terms of upregulated and downregulated DEGs from KO vs. WT at day 4 post differentiation. F The GSEA showing the expression pattern of ECM-receptor interaction-related genes in KO and WT. Fig. S3. Dip2b knockout depresses axon guidance in KSR medium. A MA plot showing the expression of DEGs from KO vs. WT at day 4 post differentiation. Upregulated and downregulated genes are plotted in red and blue, respectively. B and C KEGG pathway analysis of DEGs from KO vs. WT at day 4 post differentiation. D and E GO analysis. Top ten GO terms of upregulated and downregulated DEGs from KO vs. WT at day 4 post differentiation. F The GSEA showing the expression pattern of axon guidance-related genes in KO and WT. Table S1. The 20 most differentially expressed genes between KO vs. WT at day 8 post differentiation under N2B27 differentiation condition (Fold change ≥ 2, padj < 0.05). Table S2. The 20 most differentially expressed genes between KO vs. WT at day 4 post differentiation under N2B27 differentiation condition (Fold change ≥ 2, padj < 0.05). Table S3. The 20 most differentially expressed genes between KO vs. WT at day 9 post differentiation under KSR differentiation conditio [file 13287_2023_3482_MOESM1_ESM.docx]

Additional file 1

Loss of *Dip2b* leads to abnormal neural differentiation from mESCs

Mingze Yao, Yuanqing Pan, Tinglin Ren, Caiting Yang, Yu Lei, Xiaoyu Xing, Lei Zhang, Xiaogang Cui, Yaowu Zheng, Li Xing and Changxin Wu


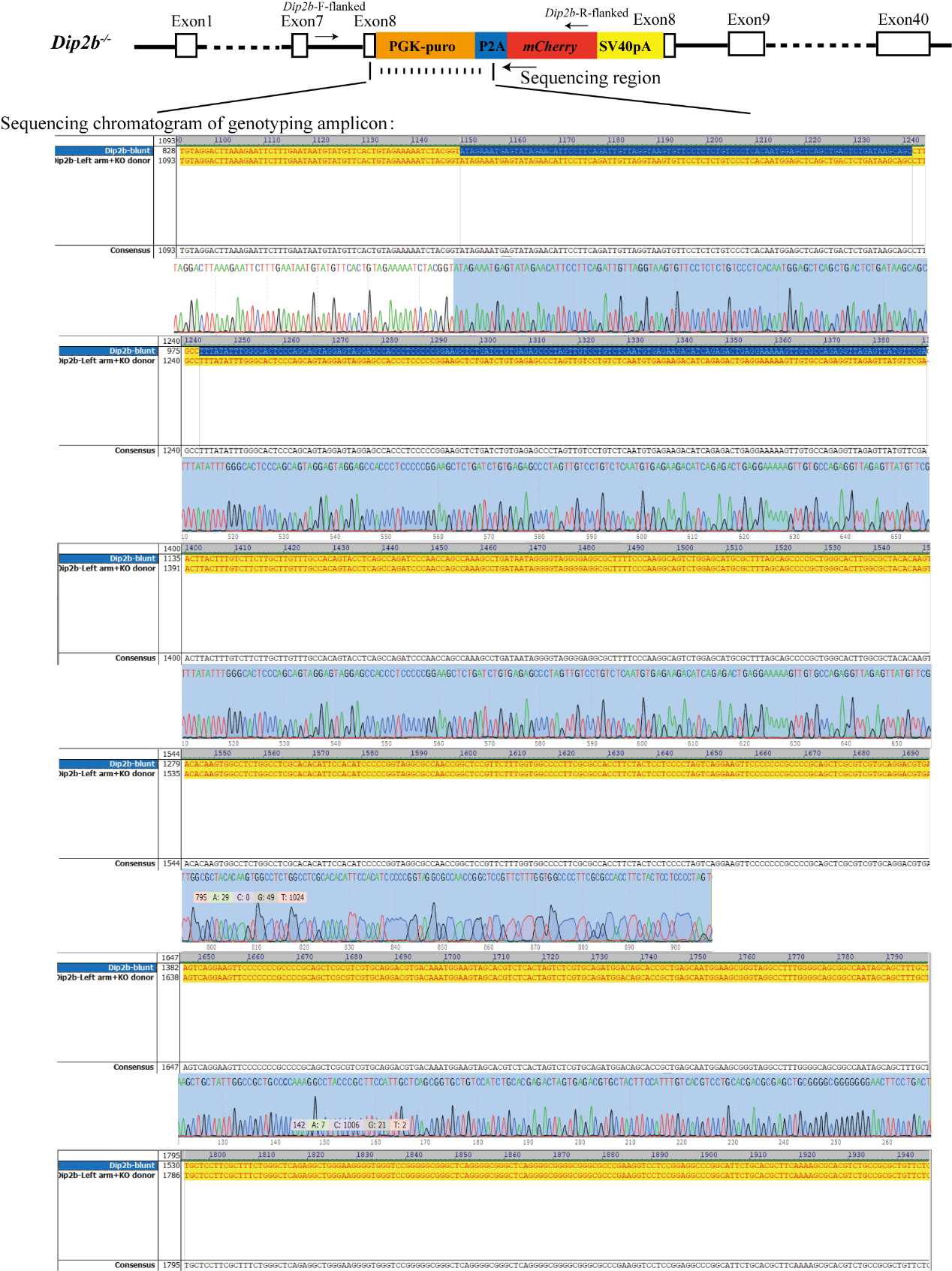


Additional file 1: Fig. S1 Verification of site-specific recombination by sequencing. A targeting vector consisting of a 1206-bp left arm, PGK-Puro, P2A-mCherry and a 1201-bp right arm and a sgRNA-PX330 plasmid were co-electroporated into 46C mESCs. The Cas9 enzyme, specifically guided by the sgRNA, can cleave the eighth exon of Dip2b to produce a double-strand break. The eighth exon of Dip2b was replaced with PGK-Puro-P2A-mCherry via homologous recombination.


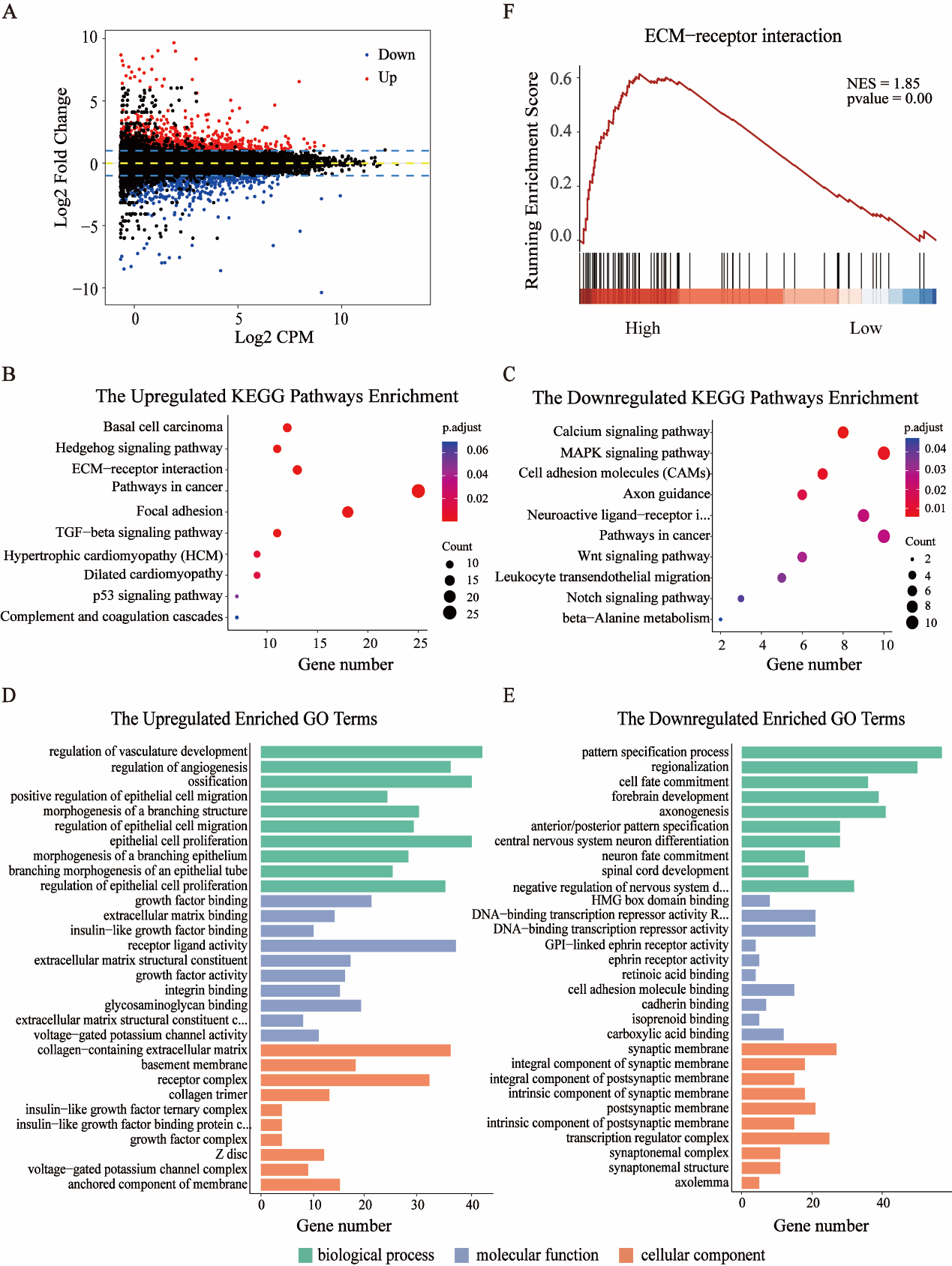


Additional file 1: Fig. S2 *Dip2b* knockout depresses axon guidance in N2B27 medium. A MA plot showing the expression of DEGs from KO vs. WT at day 4 post differentiation. Upregulated and downregulated genes are plotted in red and blue, respectively. B and C KEGG pathway analysis of DEGs from KO vs. WT at day 4 post differentiation. D and E GO analysis. Top ten GO terms of upregulated and downregulated DEGs from KO vs. WT at day 4 post differentiation. F The GSEA showing the expression pattern of ECM-receptor interaction-related genes in KO and WT.


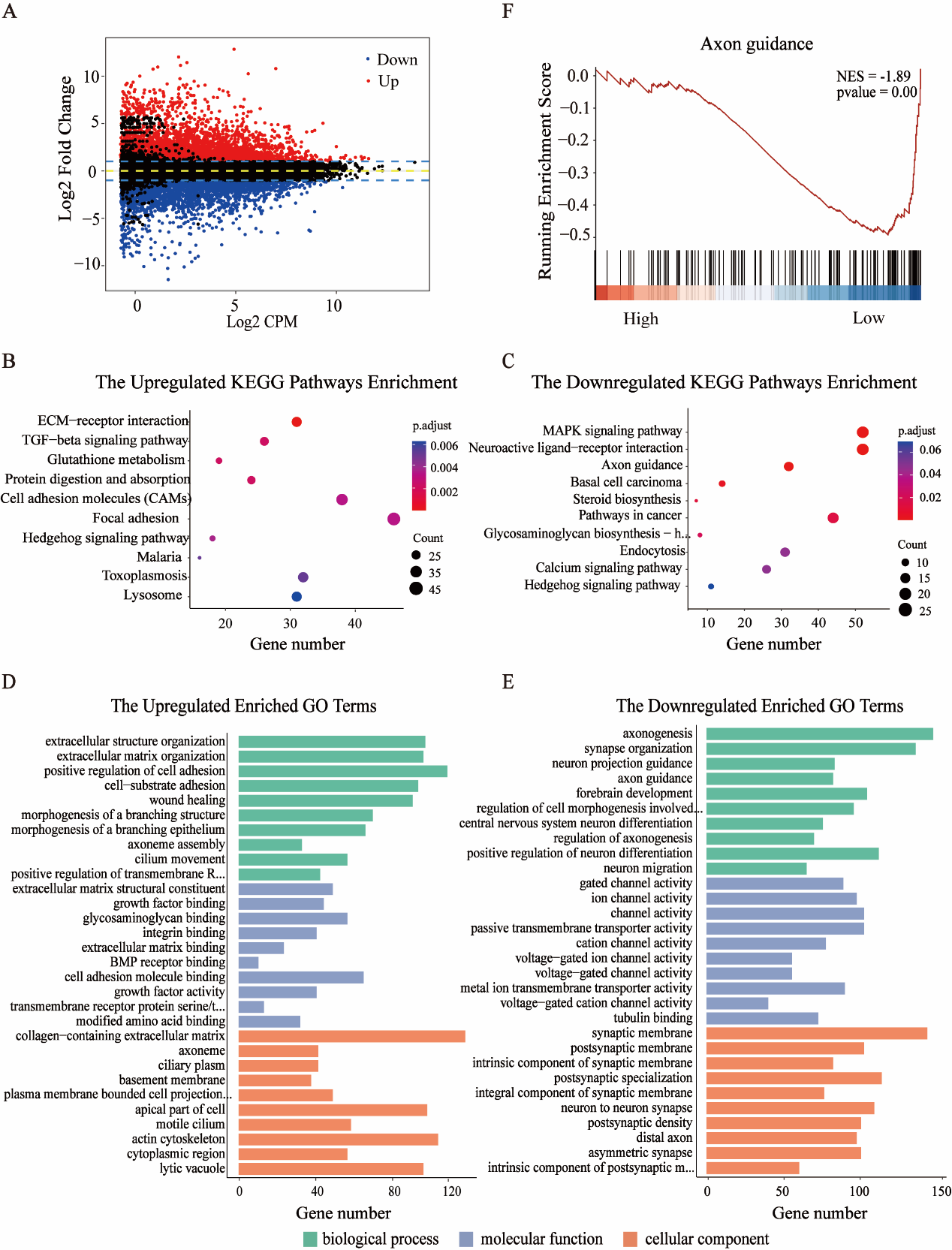


Additional file 1: Fig. S3 *Dip2b* knockout depresses axon guidance in KSR medium. A MA plot showing the expression of DEGs from KO vs. WT at day 4 post differentiation. Upregulated and downregulated genes are plotted in red and blue, respectively. B and C KEGG pathway analysis of DEGs from KO vs. WT at day 4 post differentiation. D and E GO analysis. Top ten GO terms of upregulated and downregulated DEGs from KO vs. WT at day 4 post differentiation. F The GSEA showing the expression pattern of axon guidance-related genes in KO and WT.

Additional file 1: Tables S1 The 20 most differentially expressed genes between KO vs. WT at day 8 post differentiation under N2B27 differentiation condition (Fold change ≥ 2, padj < 0.05).

| Upregulated genes | logFC | PValue | padj | Downregulated genes | logFC | PValue | padj |
| --- | --- | --- | --- | --- | --- | --- | --- |
| *Gm10359* | 12.50917 | 1.08E-86 | 2.71E-83 | *Gm49450* | -11.8804 | 1.13E-62 | 1.65E-59 |
| *Gm12671* | 12.50917 | 1.08E-86 | 2.71E-83 | *Serpinb9e* | -10.315 | 4.18E-32 | 1.42E-29 |
| *Rpl15-ps3* | 12.11825 | 8.41E-67 | 1.48E-63 | *Ttr* | -10.0599 | 3.33E-28 | 7.93E-26 |
| *Gm45234* | 10.13707 | 2.03E-28 | 5.02E-26 | *Gm20431* | -10.0473 | 4.79E-28 | 1.11E-25 |
| *Barx2* | 8.578862 | 6.97E-13 | 3.34E-11 | *Gm7332* | -9.8268 | 2.73E-29 | 7.41E-27 |
| *Eif4a-ps4* | 8.241704 | 1.42E-10 | 4.97E-09 | *Mest* | -9.76076 | 6.5E-203 | 1.2E-198 |
| *Gm15109* | 8.162203 | 4.27E-10 | 1.38E-08 | *Serpinb9g* | -9.70634 | 1.45E-23 | 2.35E-21 |
| *Gm6741* | 7.843404 | 2.21E-08 | 5.29E-07 | *Hnf1b* | -9.56572 | 8.9E-22 | 1.25E-19 |
| *Fthl17c* | 7.370833 | 2.48E-06 | 3.92E-05 | *Eif2s3y* | -9.31611 | 3.83E-19 | 4.14E-17 |
| *Gm13292* | 7.313103 | 4.54E-06 | 6.78E-05 | *Pga5* | -9.17298 | 9.21E-18 | 8.67E-16 |
| *Gm9797* | 7.225692 | 8.35E-06 | 1.16E-04 | *Serpinb9f* | -9.08385 | 9.51E-17 | 7.79E-15 |
| *Grb10* | 7.17151 | 1.63E-142 | 1.44E-138 | *Hnf4a* | -9.03787 | 2.46E-16 | 1.92E-14 |
| *Sox18* | 6.734297 | 1.38E-38 | 6.74E-36 | *Gm31135* | -9.01407 | 3.97E-16 | 3.01E-14 |
| *Gm5091* | 6.617863 | 6.67E-04 | 4.99E-03 | *Rfx6* | -8.88879 | 4.5E-15 | 2.9E-13 |
| *Gm48141* | 6.610363 | 6.67E-04 | 4.99E-03 | *Serpinb6c* | -8.66254 | 2.49E-13 | 1.25E-11 |
| *Dcstamp* | 6.503609 | 1.27E-03 | 8.60E-03 | *Cdx2* | -8.46603 | 9.6E-12 | 3.86E-10 |
| *Hus1b* | 6.243751 | 4.64E-03 | 2.48E-02 | *DXBay18* | -8.46576 | 9.6E-12 | 3.86E-10 |
| *Gm12928* | 6.160995 | 4.64E-03 | 2.48E-02 | *Gm37206* | -8.46522 | 9.6E-12 | 3.86E-10 |
| *Gm28845* | 6.110624 | 4.64E-03 | 2.48E-02 | *Rhox9* | -8.39087 | 5.71E-73 | 1.26E-69 |
| *2310043M15Rik* | 6.093862 | 4.64E-03 | 2.48E-02 | *Nxf7* | -8.26552 | 3.7E-51 | 3.64E-48 |

Additional file 1: Tables S2 The 20 most differentially expressed genes between KO vs. WT at day 4 post differentiation under N2B27 differentiation condition (Fold change ≥ 2, padj < 0.05).

| Upregulated genes | logFC | PValue | padj | Downregulated genes | logFC | PValue | padj |
| --- | --- | --- | --- | --- | --- | --- | --- |
| *Gm45837* | 9.660552 | 2.18E-23 | 9.83E-21 | *Mest* | -10.3773 | 8.66E-218 | 1.52E-213 |
| *Gm49496* | 9.028823 | 1.53E-16 | 3.28E-14 | *Peg13* | -8.6253 | 6.33E-80 | 2.23E-76 |
| *9030622O22Rik* | 8.987184 | 3.97E-16 | 7.94E-14 | *Gm49378* | -8.49058 | 3.33E-12 | 3.67E-10 |
| *Cxcl11* | 8.682997 | 1.49E-13 | 1.96E-11 | *Mid1-ps1* | -8.29158 | 8.24E-11 | 6.95E-09 |
| *Gm28901* | 8.553377 | 1.17E-12 | 1.38E-10 | *Gm49510* | -7.99996 | 3.99E-09 | 2.36E-07 |
| *Gdf1* | 8.406201 | 1.64E-11 | 1.66E-09 | *Pdcd5-ps* | -7.98306 | 3.99E-09 | 2.36E-07 |
| *Gm43118* | 8.367748 | 2.80E-11 | 2.65E-09 | *Gm42417* | -7.71071 | 1.26E-07 | 5.60E-06 |
| *Gm28539* | 8.217602 | 2.46E-10 | 1.92E-08 | *Eif2s3y* | -7.58547 | 4.11E-07 | 1.59E-05 |
| *Gm42791* | 7.85194 | 2.21E-08 | 1.15E-06 | *Gm48128* | -7.56579 | 3.94E-08 | 1.97E-06 |
| *Gm20547* | 7.725318 | 1.26E-07 | 5.60E-06 | *Gm44792* | -7.46835 | 1.36E-06 | 4.49E-05 |
| *Gm3500* | 7.533144 | 7.45E-07 | 2.68E-05 | *Srgn* | -7.3243 | 4.54E-06 | 0.00013 |
| *Gm37110* | 7.531598 | 7.45E-07 | 2.68E-05 | *Apol7e* | -7.25041 | 8.35E-06 | 0.000222 |
| *Gm44183* | 7.531598 | 7.45E-07 | 2.68E-05 | *Gm29253* | -6.82986 | 0.000187 | 0.003176 |
| *Gm3892* | 7.315075 | 4.54E-06 | 0.00013 | *Plagl1* | -6.60986 | 4.08E-91 | 1.80E-87 |
| *Gm7367* | 7.238573 | 8.35E-06 | 0.000222 | *Otp* | -6.59296 | 0.000667 | 0.009224 |
| *Gm26533* | 7.014046 | 5.33E-05 | 0.001098 | *Adgrg5* | -6.46879 | 0.001268 | 0.015195 |
| *C030014I23Rik* | 6.940771 | 9.96E-05 | 0.001891 | *Nnat* | -5.46224 | 2.46E-103 | 1.44E-99 |
| *AC240460.1* | 6.827174 | 0.000187 | 0.003176 | *G530011O06Rik* | -4.48763 | 1.09E-31 | 8.01E-29 |
| *Fthl17d* | 6.745057 | 0.000352 | 0.005431 | *Xlr5c* | -4.44158 | 5.83E-08 | 2.84E-06 |
| *Pof1b* | 6.721236 | 0.000352 | 0.005431 | *Phox2b* | -4.38396 | 1.96E-38 | 2.66E-35 |

Additional file 1: Tables S3 The 20 most differentially expressed genes between KO vs. WT at day 9 post differentiation under KSR differentiation condition (Fold change ≥ 2, padj < 0.05).

| Upregulated genes | logFC | PValue | padj | Downregulated genes | logFC | PValue | padj |
| --- | --- | --- | --- | --- | --- | --- | --- |
| *Gm45090* | 12.82748 | 4.53E-13 | 4.21E-09 | *Lhx3* | -11.468 | 1.49E-10 | 2.30E-07 |
| *Iqschfp* | 12.01278 | 1.48E-11 | 5.50E-08 | *Gm37206* | -10.2923 | 2.06E-08 | 8.32E-06 |
| *Gm44170* | 11.42061 | 1.83E-10 | 2.62E-07 | *Mrip-ps* | -10.1338 | 3.74E-08 | 1.29E-05 |
| *Gdf1* | 11.12719 | 6.32E-10 | 6.85E-07 | *B3galt5* | -10.0886 | 4.62E-08 | 1.52E-05 |
| *Gm6158* | 10.94541 | 1.35E-09 | 1.18E-06 | *Lamp5* | -9.5812 | 5.48E-12 | 2.55E-08 |
| *Gm29216* | 10.78442 | 5.02E-17 | 9.35E-13 | *Gm12928* | -9.36435 | 8.52E-07 | 0.000144 |
| *A830029E22Rik* | 10.73032 | 3.31E-09 | 1.91E-06 | *Gm10705* | -9.21521 | 1.52E-06 | 0.000219 |
| *Zscan4-ps2* | 10.23647 | 2.49E-08 | 9.87E-06 | *Gm9762* | -9.16406 | 1.91E-06 | 0.000257 |
| *1700012P22Rik* | 9.993921 | 6.89E-08 | 2.14E-05 | *Gm14344* | -9.07886 | 2.63E-06 | 0.00032 |
| *Plac9b* | 9.720421 | 2.08E-07 | 5.03E-05 | *Tal1* | -9.02234 | 4.89E-12 | 2.55E-08 |
| *Gm45234* | 9.445439 | 6.10E-07 | 0.000117 | *Klhl14* | -8.98125 | 3.70E-06 | 0.000397 |
| *Nek5* | 9.342653 | 9.13E-07 | 0.000149 | *Gm15429* | -8.88056 | 5.29E-06 | 0.00053 |
| *Iqcj* | 9.239302 | 1.41E-06 | 0.000208 | *Gm37303* | -8.83834 | 6.37E-06 | 0.000597 |
| *Gm6565* | 9.169261 | 1.77E-06 | 0.000243 | *Kcna3* | -8.78502 | 7.73E-06 | 0.000683 |
| *Naa11* | 9.100489 | 2.24E-06 | 0.00029 | *Gm38057* | -8.75802 | 8.53E-06 | 0.000731 |
| *Gm43126* | 9.072929 | 2.63E-06 | 0.00032 | *Slc6a5* | -8.71455 | 2.21E-10 | 2.93E-07 |
| *Fthl17c* | 9.041278 | 2.86E-06 | 0.000339 | *Fam205c* | -8.70115 | 1.04E-05 | 0.000841 |
| *Pifo* | 9.03832 | 2.86E-06 | 0.000339 | *1700109K24Rik* | -8.61211 | 1.43E-05 | 0.001029 |
| *Cldn9* | 9.036601 | 3.76E-11 | 8.75E-08 | *Glra1* | -8.58857 | 3.75E-10 | 4.65E-07 |
| *Gm53* | 8.950262 | 4.03E-06 | 0.000419 | *Pcdhac2* | -8.56034 | 1.79E-05 | 0.001224 |

Additional file 1: Tables S4 The 20 most differentially expressed genes between KO vs. WT at day 4 post differentiation under KSR differentiation condition (Fold change ≥ 2, padj < 0.05).

| Upregulated genes | logFC | PValue | padj | Downregulated genes | logFC | PValue | padj |
| --- | --- | --- | --- | --- | --- | --- | --- |
| *Gm11237* | 12.47796 | 2.02E-12 | 1.88E-08 | *Tfap2b* | -10.121 | 4.06E-08 | 9.45E-06 |
| *Obox4-ps12* | 12.13779 | 8.66E-12 | 3.22E-08 | *Mest* | -9.89881 | 5.30E-16 | 9.86E-12 |
| *Gm428* | 11.32552 | 2.70E-10 | 7.17E-07 | *Gm45837* | -9.63327 | 2.75E-07 | 4.10E-05 |
| *Fthl17c* | 11.24985 | 3.75E-10 | 7.76E-07 | *Gm45062* | -9.08435 | 2.43E-06 | 0.000223 |
| *Spopfm3* | 10.90333 | 1.61E-09 | 2.14E-06 | *Hoxa4* | -8.25257 | 5.36E-05 | 0.002889 |
| *Arl14epl* | 10.8709 | 1.83E-09 | 2.26E-06 | *Gm44503* | -8.2254 | 6.13E-05 | 0.00325 |
| *Gm9343* | 10.63172 | 4.98E-09 | 2.92E-06 | *Pagr1a* | -8.09075 | 9.36E-05 | 0.004609 |
| *BC080695* | 10.48811 | 9.10E-09 | 3.91E-06 | *Slc17a6* | -7.95991 | 0.000147 | 0.006564 |
| *Gm44739* | 10.47728 | 9.40E-09 | 3.91E-06 | *Gm7367* | -7.95356 | 0.000147 | 0.006564 |
| *Pramef25* | 10.40495 | 1.28E-08 | 4.86E-06 | *Otp* | -7.81369 | 0.000241 | 0.009885 |
| *Gm4294* | 10.3538 | 1.59E-08 | 5.37E-06 | *Plagl1* | -7.71259 | 8.55E-12 | 3.22E-08 |
| *B020031M17Rik* | 10.29586 | 1.98E-08 | 5.67E-06 | *Nnat* | -7.64495 | 6.17E-12 | 3.22E-08 |
| *Frg2f4* | 10.09944 | 4.42E-08 | 9.80E-06 | *Gm13425* | -7.64187 | 0.00041 | 0.015396 |
| *Gm13078* | 10.07825 | 4.82E-08 | 1.00E-05 | *Nxph1* | -7.40079 | 0.000901 | 0.028789 |
| *BB287469* | 10.01348 | 6.29E-08 | 1.21E-05 | *Lrrn3* | -7.40078 | 0.000901 | 0.028789 |
| *Obox4-ps14* | 9.966572 | 7.56E-08 | 1.41E-05 | *Prdm13* | -7.17981 | 0.00175 | 0.047379 |
| *Gm13083* | 9.926368 | 8.72E-08 | 1.53E-05 | *Zfp968-ps* | -6.79394 | 0.000494 | 0.018101 |
| *Gm6189* | 9.801021 | 3.75E-10 | 7.76E-07 | *Peg13* | -6.68376 | 1.06E-08 | 4.09E-06 |
| *Magea4* | 9.716058 | 2.08E-07 | 3.34E-05 | *Sp8* | -6.55233 | 2.40E-09 | 2.31E-06 |
| *Gm8723* | 9.652955 | 2.60E-07 | 3.97E-05 | *Angpt1* | -6.51467 | 3.64E-08 | 8.79E-06 |

Additional file 1: Tables S5 PCR primer sequences selected for validation

| Gene | Primer Sequences (5’-3’) |
| --- | --- |
| Left arm (Donor sequencing) | AGCTATGACCATGATTACTGTGCCGACTCTATAAAGAGTC |
|  | CCTCCCCTACCCCTATTATCAGGCTTTGGCTGGTTGGGATCTG |
| PGK-puro -P2A-mcherry (Donor sequencing) | GCCTGATAATAGGGGTAGGGGAGGCGCTTTTCCCAAG |
|  | GGTTTCCCCGTCGCAGTGAAAAAAATGCTTTATTTGTG |
| Right arm (Donor sequencing) | GCATTTTTTTCACTGCGACGGGGAAACCAGTCTACACTCTCAC |
|  | GTAAAACGACGGCCAGTGCCAATTGCCTTTCCTTCCGATGTGAAATG |
| *Dip2b* (Homozygote verified sequencing) | ACCTCAGCCAGATCCCAACC |
|  | CACTTGGCTTGGGTAGAGCC |
| *Dip2b* (Heterozygote verified sequencing) | ACCTCAGCCAGATCCCAACC |
|  | CACTTGGCTTGGGTAGAGCC |
| *Dip2b* (Dip2b-flanked) | CAAGCCCGGTGCCGCCACTAACTTCAGCTTGTTGAAGCAG |
|  | CCCCCAAGCAGCTTCTCAGCCATTTCAAAG |

Additional file 1: Tables S6 qRT-PCR primer sequences selected for validation

| Gene | Primer Sequences (5’-3’) |
| --- | --- |
| *Dip2b* | ACCTCAGCCAGATCCCAACC |
|  | CACTTGGCTTGGGTAGAGCC |
| *Gapdh* | AACTTTGGCATTGTGGAAGGGCTCA |
|  | TTGGCAGCACCAGTGGATGCAGGGA |
| *Nanog* | CTCAAGTCCTGAGGCTGACA |
|  | TGAAACCTGTCCTTGAGTGC |
| *Oct4* | TAGGTGAGCCGTCTTTCCAC |
|  | GCTTAGCCAGGTTCGAGGAT |
| *Sox2* | CTGCAGTACAACTCCATGACCAG |
|  | GGACTTGACCACAGAGCCCAT |
| *Klf4* | AACATGCCCGGACTTACAAA |
|  | TTCAAGGGAATCCTGGTCTTC |
| *Gata4* | CCCTACCCAGCCTACATGG |
|  | ACATATCGAGATTGGGGTGTCT |
| *Sox17* | CGAGCCAAAGCGGAGTCTC |
|  | TGCCAAGGTCAACGCCTTC |
| *Gsc* | ACCATCTTCACCGATGAGCAGC |
|  | CTTGGCTCGGCGGTTCTTAAAC |
| *T* | GCTTCAAGGAGCTAACTAACGAG |
|  | CCAGCAAGAAAGAGTACATGGC |
| *Pax6* | GCAGATGCAAAAGTCCAGGTG |
|  | CAGGTTGCGAAGAACTCTGTTT |
| *Nestin* | CCCTGAAGTCGAGGAGCTG |
|  | CTGCTGCACCTCTAAGCGA |
| *Neurod1* | ATGACCAAATCATACAGCGAGAG |
|  | TCTGCCTCGTGTTCCTCGT |
| *β-Ⅲ Tubulin* | ACTTGGAACCTGGAACCATGG |
|  | GGCCTGAATAGGTGTCCAAAGG |
| *Map2* | Agcactgattgggaagcact |
|  | caattcaaggaagttgtaaagtagtgaagtTTg |
| *Sox1* | GCACACAGCGTTTTCTCGG |
|  | ACATCCGACTCCTCTTCCC |
| *Dcc* | GCTATGGTGTTGGCAGTCCT |
|  | CGGGGTCAGTGGGATCTGTT |
| *Robo3* | ATAGAGGCCTTCAGCCAAGC |
|  | TCCTGGGTTTGAACAGGCTC |
| *Epha7* | AACCGGGAACAGTGTACGTC |
|  | GGAGACTGCTGTCGCTTCAA |
| *L1cam* | AGGGTGGCAAATACTCAGTGAA |
|  | GCTGCCAAAGGCCTTCTCTT |
| *Lrr4c* | TGCTGTTGCTGAAGGATCAA |
|  | ACACTTCATTTGGTTTCTTCTCAG |
| *Unc5d* | GTGGTGGTCTACGTGGATGG |
|  | GCAGAGACCATCTGTGCAGT |
| *Npnt* | CGGGCCAAACAAGTGCAAAT |
|  | ACACGATAGGGCACTTGAGC |
| *Colla2* | CCAAGGGTGCTACTGGACTC |
|  | GCTCACCCTTGTTACCGGAT |
| *Itga8* | ACACGTTCCTCAAGAGAAAGAA |
|  | GGAGTGGCCCAAATAACCGA |
| *Epha3* | GAGGTCAAATACTATGAAAAGGAGC |
|  | AATTTGCGGCTGTTGGTTCC |
| *Cxcr4* | CATGGAACCGATCAGTGTGAG |
|  | TGAAGGCCAGGATGAGAACG |
| *Gsk3b* | GCTGTGTGTTGGCTGAATTGT |
|  | TGCTCCTGGTGAGTCCTTTGT |
| *Vtn* | CAGTGCAAGCCCCAAGTAAC |
|  | CGTCCGTCCGAGGATTTAGG |
| *Thbs2* | GAGAGCCAGTCCGATGTCTG |
|  | TCTTCACGTGACCTGGTGTG |
