# Supplementary material for: Loss of Dip2b leads to abnormal neural differentiation from mESCs
Source: Stem Cell Res Ther. 2023 Sep 13;14:248. doi: 10.1186/s13287-023-03482-6 (PMC10500737; doi:10.1186/s13287-023-03482-6)
Supplement: Supplementary file 2 — Additional file 2. Uncropped gel images. Uncropped gel images are attached. Images used in the main figure are marked in red squares. Deletion region was not detectable at DNA level by PCR in the Dip2b gene knockout cell line. [file 13287_2023_3482_MOESM2_ESM.docx]

Additional file 2

Loss of *Dip2b* leads to abnormal neural differentiation from mESCs

Mingze Yao, Yuanqing Pan, Tinglin Ren, Caiting Yang, Yu Lei, Xiaoyu Xing, Lei Zhang, Xiaogang Cui, Yaowu Zheng, Li Xing and Changxin Wu


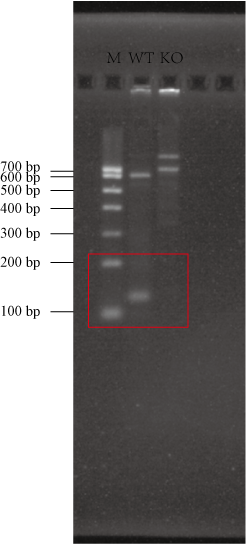


Additional file 2: Uncropped gel images. Uncropped gel images are attached. Images used in the main figure are marked in red squares. Deletion region was not detectable at DNA level by PCR in the *Dip2b* gene knockout cell line.
